# Death of Prof. Samuel D. Gross

**Published:** 1884-06

**Authors:** 


### Editorial.

### DEATH OF PROF. SAMUEL D. GROSS.

The profession is again shocked at the announcement of the death of one of its most brilliant and beloved members. Prof. S. D. Gross died on the 6th of May, at his home in Philadelphia, surrounded by family and friends, at the ripe old age of 79 years. With perhaps the single exception of the late J. Marion Sims, Dr. Gross was more widely and favorably known and his death will be more regretted and mourned throughout the civilized world than would be that of any member of the profession in America. The following brief synopsis from the *Medical Record*, of the labors of this great and good man, with the honors conferred upon him by the most prominent institutions of learning in the civilized world, will be read with pride by every member of the profession:

The life-work of this great man is finished, and how magnificent that work has been! Rather should we say, how magnificent it is, for though 'tis finished, and the great mind which wrought it has ceased to be, the perfected work will live on till there is no longer a place for the art of medicine. Even while we write there is sorrow in the profession throughout the length and breadth of our land; and wherever science is known, and greatness and learning honored, his death will be deplored.

Samuel D. Gross, M. D., LL. D., D. C. L. Oxon., LL. D. Cantab., LL. D. Edinb., died on Tuesday, May 6th, at his residence in Philadelphia, after an illness of some weeks. He was born near Easton, Pa., in July, 1805, and was therefore in the seventy-ninth year of his age. He received his classical education at Wilkesbarre, and at the High School at Lawrenceville, N. J., and began his medical studies at an early age, under the preceptorship of Dr. J. K. Swift, of Easton, after which he continued them for nearly two years under the celebrated Dr. George McClellan, of Philadelphia. He was graduated from the Jefferson Medical College in 1828, and entered upon practice in Philadelphia.

The leisure hours which fall to the lot of every young practitioner were spent by Dr. Gross in the translation of several standard French and German works. But his ability and activity removed him above the plane of the translator, and two years after graduating he brought out his first original work upon "Diseases and Injuries of the Bones and Joints." At this time he removed to Easton, but was elected in 1833 as Demonstrator of Anatomy in the Medical College of Ohio. This position he accepted, and two years later was elected Professor of Pathological Anatomy in the Medical Department of the College in Cincinnati. Here he delivered the first systematic course of lectures on pathological anatomy ever given in the United States, writing meanwhile his second book, "The Elements of Pathological Anatomy," the first work of its kind published in this country. From this chair he was called to the Chair of Surgery in the University of Louisville, where for ten years he gave evidences of the genius which was subsequently to be honored by the civilized world. From this chair he was called to that of Surgery in the University of New York, but returned at the end of one year, at the earnest solicitations of his former colleagues. Here he remained until 1856, when his Alma Mater called him to teach in the halls whence he had gone forth as a distinguished student.

Shortly after coming to Philadelphia he founded the Pathological Society of Philadelphia, being its first president. In 1867 he was elected President of the American Medical Association, and four years later was chosen Chairman of the Teachers' Medical Convention in Washington. In 1872 he visited Europe for the second time, not as an unknown or a rising man, but as a master in his science and art, a successful surgeon, and an author, whose reputation had circled the globe. While in England, the University of Oxford celebrated its one thousandth anniversary, and gracefully complimented the great surgeon and American medicine by conferring upon Dr. Gross the degree of D. C. L. In 1880 the University of Cambridge conferred upon him the degree of LL. D., which degree he had already received from the Jefferson College. On April 1[illegible], 1884, the University of Edinburgh, at its tercenary anniversary, conferred the degree of LL. D. upon him, and the University of Pennsylvania paid the same tribute to his learning on May 1st.

Not the least among his honors was his unanimous election to the presidency of the International Medical Congress, which met in Philadelphia in 1876. In 1880 he organized the American Surgical Association, of which he was president until 1883.

Of his greatest literary work, his "System of Surgery," it were scarcely necessary to speak. While his fame goes down to the posterity of succeeding generations as a blessed heritage, his great work on surgery will remain a tangible legacy to the students of many lands and tongues.

In four great cities Dr. Gross has been a teacher of surgery, and thousands of his pupils are scattered throughout the Union. As a teacher of surgery he has long been recognized as the greatest which the country has ever produced.

At a dinner given to him in Philadelphia, in April, 1879, Dr. Gross said:—"After fifty years of earnest work I find myself still in the harness; but although I have reached that age when most men, tired of the cares of life, seek repose in retirement and abandon themselves to the study of religion, the claims of friendship, or the contemplation of philosophy, my conviction has always been that it is far better for a man to wear out than to rust out. Brain work, study, and persistent application, has been a great comfort to me, as well as a great help; it has enhanced the enjoyment of daily life, and added largely to the pleasures of the lecture-room and of authorship; indeed, it will always, I am sure, if wisely regulated, be conducive both to health and longevity. A man who abandons himself to a life of inactivity, after having always been accustomed to work, is practically dead."

How truly he carried out these precepts is seen by the fact that, within a few weeks of his death, he has prepared two able papers—one on "Wounds of the Intestines," for the American Surgical Association, which met in Washington last week; the other on the "Lacerations of the Female Sexual Organs," for the Obstetrical section of the American Medical Association, which met in the same city during the present week. Though well-nigh fourscore years of age, he has never allowed the great mind which has guided the surgical world to become for one moment idle.

As a companion and as a host Dr. Gross was one of the most genial and generous of men, and few who ever heard his voice will forget its majestic power and sweetness. As a writer he was voluminous.

In 1843 he published "An Experimental and Critical inquiry into the Nature and Treatment of Wounds of Intestines, and it is a curious coincidence that just forty-one years afterward he should contribute a paper on this subject; in 1851, "A Practical Treatise on the Diseases, Injuries and Malformations of the Bladder;" in 1854, "A Practical Treatise on Foreign Bodies in the Air Passages," and the same year he issued a "History of Kentucky Surgery." In 1859 he published his noblest work, "A System of Surgery, Pathological, Diagnostic, Therapeutic, and Operative," the sixth edition of which was put out in 1882. At the outbreak of the war Dr. Gross issued a "Manual of Military Surgery," which passed through two editions and afforded important service in fitting young military surgeons for the better and more efficient discharge of their duties on the field and in the hospital. In 1861 he edited a large volume entitled "Lives of Eminent Physicians and Surgeons of the Nineteenth Century." In 1876 he published a "History of American Medical Literature from 1776 to the Present Time," and the same year an elaborate paper entitled "A Century of American Surgery."

In addition to the comprehensive standard works already mentioned, Dr. Gross also made many other noteworthy contributions to the literature of the medical profession, chiefly in the form of monographs and miscellaneous papers, contained in the current medical press of the country.

Dr. Gross leaves four children, upon one of whom, Professor Samuel W. Gross, now gracefully rests the mantle so long worn by his distinguished father, as Professor of Surgery in Jefferson College.

In addition to his numerous titles from American and British institutions, he was member or fellow of several foreign societies, including the British Medical Association, the Imperial Medical Society of Vienna, the Royal Medico-Chirurgical Society of London, and the Clinical and Pathological Societies of London.

Dr. Gross was the first to suggest and perform the operation of wiring the dislocated clavicle to the sternum, or acromion process; the suturing of divided nerves and tendons; deep stitches for wounds of the abdomen; the direct operation for the radical cure of hernia by suturing the pillars of the ring; an operation for the cure of neuralgia in old persons, and a modification of Pirogoff's operation; and was the first to describe prostatorrhœa. Eminence in medicine, whether as an art or science, requires labor which demands the most untiring industry, and a high order of talent. In neither of these requisites was he wanting, and whether progress in medicine be regarded as the history of the profession or the development of the curative art, it would be impossible to omit the history of his untiring and fruitful labors. Profoundly learned in all the anatomical, medical, and philosophical lore of his own and and former times, there was lacking in him no quality requisite for an encyclopædic writer, whether in the literary or professional world. Of him, as of the father of modern medicine, it may be said that, "finding medical science confounded under a multitude of dogmatic systems, he appears to have made it his object to reform these evils, to reconcile scientific requirements and practical skill, to bring back the unity of medicine as it had been understood by Hippocrates, and at the same time to raise the dignity of medical practitioners."

There are epochs in the history of medicine with which famous and undying names are inseparably associated, and there are great names belonging to special departments in medicine. But for Dr. Gross, no one great operation is called by his name, nor was it his choice to make his own limits in the great field of medicine. His fame will rest securely on that highest work of having guided the current of medical science into new channels, and leading it into more fruitful fields by directing attention to the internal and real conditions of disease. His introduction of the study of morbid anatomy into this country makes him the bridge which spans the chasm between the epochs of the exclusive study of symptoms and the latter efforts to find the cause of diseases by thorough scientific study. In his life was summed up the progress of medical learning, the elevation of his profession, and the extension of the limits of medical knowledge.

